# Cross-talk between Human Papillomavirus Oncoproteins and Hedgehog Signaling Synergistically Promotes Stemness in Cervical Cancer Cells

**DOI:** 10.1038/srep34377

**Published:** 2016-09-28

**Authors:** Kanchan Vishnoi, Sutapa Mahata, Abhishek Tyagi, Arvind Pandey, Gaurav Verma, Mohit Jadli, Tejveer Singh, Sukh Mahendra Singh, Alok C. Bharti

**Affiliations:** 1 Molecular Oncology Laboratory, Department of Zoology, University of Delhi, Delhi, India; 2 Division of Molecular Oncology, Institute of Cytology & Preventive Oncology (ICMR), Noida, Uttar Pradesh, India; 3 School of Biotechnology, Banaras Hindu University, Varanasi, Uttar Pradesh, India; 4 Molecular Oncology Laboratory, B.R. Ambedkar Center for Biomedical Research (ACBR), University of Delhi, New Delhi, India

## Abstract

Viral oncoproteins E6/E7 play key oncogenic role in human papillomavirus (HPV)-mediated cervical carcinogenesis in conjunction with aberrant activation of cellular signaling events. GLI-signaling has been implicated in metastasis and tumor recurrence of cervical cancer. However, the interaction of GLI-signaling with HPV oncogenes is unknown. We examined this relationship in established HPV-positive and HPV-negative cervical cancer cell lines using specific GLI inhibitor, cyclopamine and HPVE6/E7 siRNAs. Cervical cancer cell lines showed variable expression of GLI-signaling components. HPV16-positive SiHa cells, overexpressed GLI1, Smo and Patch. Inhibition by cyclopamine resulted in dose-dependent reduction of Smo and GLI1 and loss of cell viability with a higher magnitude in HPV-positive cells. Cyclopamine selectively downregulated HPVE6 expression and resulted in p53 accumulation, whereas HPVE7 and pRb level remained unaffected. siRNA-mediated silencing of HPV16E6 demonstrated reduced GLI1 transcripts in SiHa cells. Cervical cancer stem-like cells isolated by side population analysis, displayed retention of E6 and GLI1 expression. Fraction of SP cells was reduced in cyclopamine-treated cultures. When combined with E6-silencing cyclopamine resulted in loss of SP cell’s sphere-forming ability. Co-inhibition of GLI1 and E6 in cervical cancer cells showed additive anti-cancer effects. Overall, our data show existence of a cooperative interaction between GLI signaling and HPVE6.

Cervical cancer is the leading cause of cancer-related deaths in women of underdeveloped region globally^1^. Several large scale clinico-epidemiological and molecular studies have established that persistent infection of high risk human papillomaviruses (HR-HPV) plays the etiological role in cervical carcinogenesis^2^. Almost all cervical tumors show presence of HPV-infection irrespective of their stage or grade, which is indicative of an essential contributory role of infected HPV genome from tumor initiation till late stages of carcinogenic progression^3^. HR-HPV encoded oncoproteins, E6 and E7 operate cell-immortalization and transformation through degradation or inactivation of key cell cycle regulatory proteins p53 and pRb, respectively^4^,^5^. Among 15 different HR-HPV known to infect genital mucosa, HPV16 and 18 are the two most predominant types that collectively contribute up to 70–90% of cervical malignancies^6^ and represent a formidable challenge to women health. Most of the affected women report this malignancy at a very advance stage^7^. Alike, other cancers, the treatment response in late stage cervical malignancies is poor accompanied by emergence of chemo/radioresistance and tumor recurrences leading to patient mortality^8^. HPV oncogenes E6 and E7 have a differential role in promoting chemoresistance in cervical cancer cells^9^. Though, several attempts to develop anti-HPV and anti-cancer agents have been made but none of them could emerge as clinical reality^10^. Therefore, further understanding of host-virus interactions specifically the one that governs the behavior of cervical cells at later stages of malignancy is necessary to develop novel therapeutic strategies.

The hedgehog (Hh)/GLI signaling, which is a highly conserved pathway that regulates patterning and progenitor cell fate in normal animal development, has been implicated in promoting stemness, chemoresistance^11^,^12^,^13^ and metastasis^14^,^15^,^16^,^17^. The key events in activation of GLI signaling are binding of ligand Ihh, Shh to its receptor Patch, which relieves its inhibition on another receptor Smo. Active Smo results in activation of GLI transcription factor, GLI1, GLI2 and GLI3 where GLI1 act as main activator form of the GLI signaling pathway^18^. Nevertheless, aberrant activation of GLI signaling is a key feature of many cancers^18^,^19^.

Presence of components of the hedgehog signaling in advance stages of the cervical cancer^20^,^21^,^22^ and cervical cancer cell lines^23^ suggest an active involvement of hedgehog signaling in development of cervical carcinogenesis and its association with recurrence and onset of chemoresistance. However, possible interaction of HPV oncogene with this key signaling pathway in cervical cancer is not deciphered clearly. Thus, in present study, we used established cervical cancer cell lines to explore interaction of HPV E6 oncoprotein in activation of Hedgehog signaling using a specific inhibitor against Smo, cyclopamine (Cyc) and specific siRNA against HPV E6 and E7 oncogenes. We found that HPV oncoprotein E6 and GLI1, downstream of Hedgehog signaling pathway act in coordination in cervical cancer cell survival and get overexpressed in cervical cancer stem cells. Moreover, simultaneous inhibition of both HPV E6 and GLI can specifically target cancer stem-like cells.

## Results

### Active GLI signaling in cervical cancer cell lines

**Figure 1:**
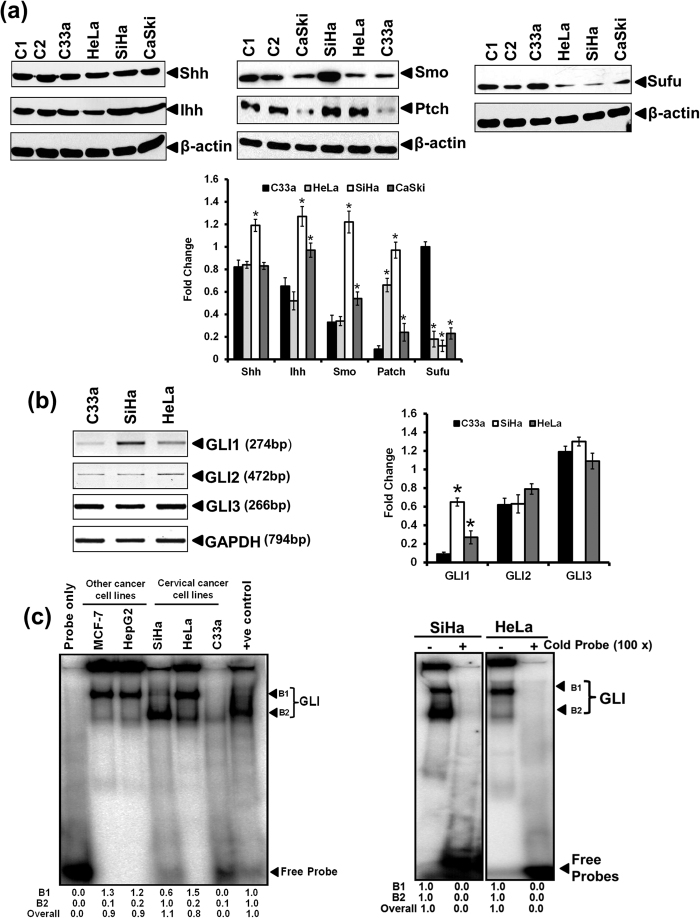
Level of key components of Hedgehog/GLI pathway and GLI activity in cervical cancer cell lines. (**a**) Representative immunoblots of total cellular proteins (50 μg/lane) from different cancer cell line (C1: MB-MDA-231, C2: HaCaT) tested for the expression of Shh, Ihh, Smo, Patch and Sufu. The blots were stripped and reprobed for β-actin and evaluated as input control. (a, lower panel). Aggregated mean (±S.D.) abundance ratios of the band intensity of indicated proteins in cervical cancer cells normalized to β-actin in three independent experiments. *p value ≤ 0.05 versus expression of the respective proteins in control HPV-negative C33a cells. **(b)** Evaluation of transcript level of GLI1, GLI2 and GLI3 by RT-PCR. Representative photograph of EtBr-stained 2% agarose gel showing PCR amplification of different GLI members in cDNA prepared from total RNA of different cervical cancer cells. GAPDH was used as input control for normalization. (b, right panel) Aggregated mean (±S.D.) abundance ratios of the GLI1, GLI2 and GLI3 transcripts w.r.t. GAPDH in three independent experiments. *p value ≤ 0.05 versus expression of respective GLI member in HPV negative C33a cells. **(c)** Evaluation of GLI-specific DNA-binding activity in cervical cancer cell lines. Representative autoradiogram showing GLI-specific binding and gel retardation of ^32^P-labeled oligonucleotide probe having GLI consensus sequence when incubated with nuclear proteins (10 μg/lane) isolated from different cervical and other cell lines and run on non-denatured gel. (c, right panel) Binding specificity was ascertained in a competition assay performed with nuclear extracts of SiHa and HeLa cells incubated with unlabelled 100 molar excess of specific competitor GLI probe. Fold change was calculated following densitometric evaluation (B1: Band First, B2: Band second).

The expression pattern of GLI signaling components was tested in established cervical cancer cell lines, C33a, SiHa, HeLa and CaSki along with other cancer cells MDA-MB-231 (breast cancer) and HaCaT (immortalized Keratinocytes) that were used as control (Fig. 1a). Key components of GLI signaling, ligand (Shh, Ihh), receptor (Patch, Smo), and negative regulator (Sufu) were expressed at protein level in all cervical cancer cells. Our attempt to detect GLI protein failed (data not shown). However, transcript level of GLI1, GLI2 and GLI3 were detectable in all the cell lines albeit at different level (Fig. 1b). Shh and Ihh were the most abundant proteins [Fig. 1a (left panel)]. The expression of Patch was higher in SiHa and HeLa compared to CaSki and C33a cells. Smo was expressed by all cell lines but its level was higher in HPV16-positive SiHa cells [Fig. 1a (middle panel)]. Sufu level was comparatively low, typically in HPV-positive, SiHa, HeLa and CaSki cells [Fig. 1a (right panel)]. Semi-quantitative analysis of GLI1, GLI2 and GLI3 in cervical cancer cells demonstrated elevated levels of GLI1 transcripts particularly in HPV16-positive SiHa cells. However, there was no difference in transcript level of GLI2 and GLI3 in cervical cancer cells (Fig. 1b). Transcript level of GLI3 was highest among all GLI isoforms. Further examination of GLI DNA-binding activity as assessed by EMSA, revealed presence of two discrete retarded bands and composition of signals in each band varied with cell type (Fig. 1c). Consistent two bands were observed in EMSA and both were considered to represent GLI DNA-binding activity. High GLI binding activity was observed in HeLa and SiHa cells whereas GLI DNA binding activity was detectable only at low level in C33a cells.

### Inhibition of GLI signaling by cyclopamine mediate loss of cervical cancer cell viability

**Figure 2:**
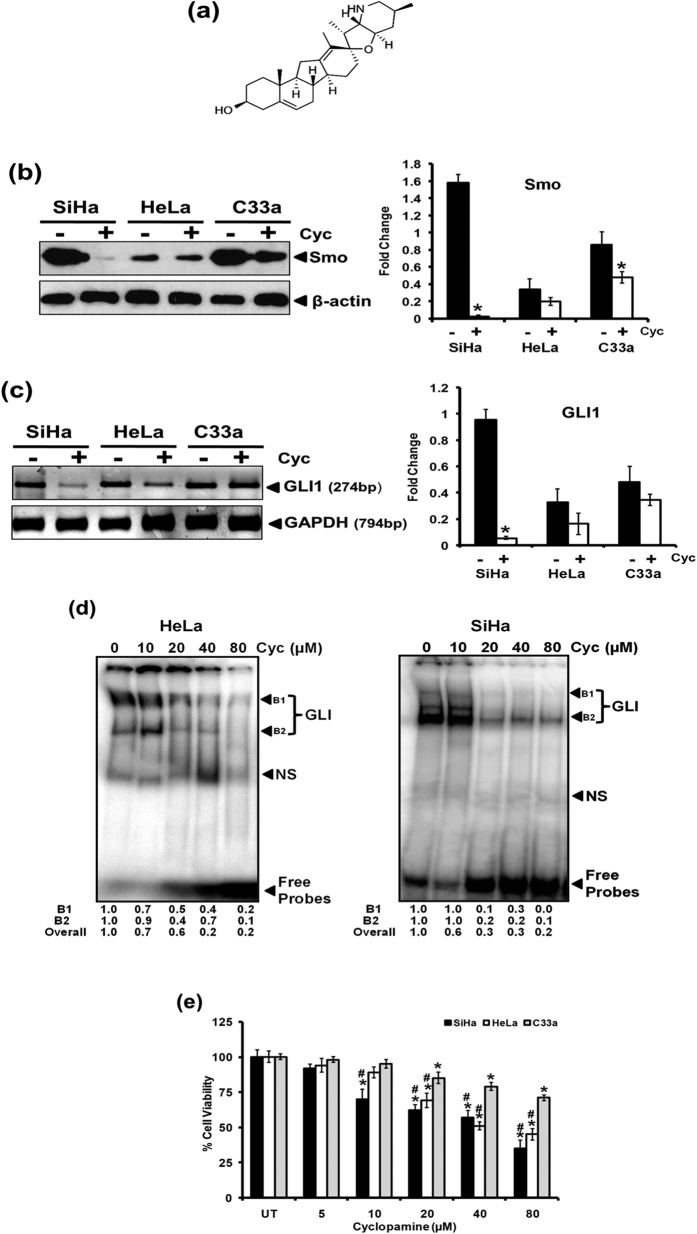
Effect of cyclopamine on GLI signaling and cell viability in cervical cancer cell lines. **(a**) Structure of cyclopamine. **(b,c)** Effect of cyclopamine on expression of its specific molecular target Smo **(b)** and downstream mediator of GLI signaling, GLI1 **(c)**. **(b)** Representative immunoblots showing Smo expression in cellular proteins (50 μg/lane) from cervical cancer cell lines treated with cyclopamine (20 μM) for 24 h and evaluated by SDS-PAGE followed by immunoblotting as indicated in Methods. Blots were striped and reprobed for β-actin as input control. **(b,** right panel) Aggregated mean (±S.D.) abundance ratios of the band intensity of Smo proteins in cervical cancer cells normalized to β-actin in three independent experiments. *p value ≤ 0.05 versus untreated control cells. Band intensity of β-actin was used as control for densitometric analysis. **(c)** Representative photograph of EtBr-stained 2% agarose gel showing amplification of GLI1 in cDNA prepared from cyclopamine (20 μM) treated and untreated cervical cancer cells using RT-PCR. GAPDH was used as input control for normalization. **(c,** right panel) Aggregated mean (±S.D.) abundance ratios of the GLI1 transcripts w.r.t. GAPDH in three independent experiments. *p value ≤ 0.05 versus untreated control cells. **(d)** Effect of cyclopamine on DNA-binding activity of GLI. Representative autoradiogram showing gradual loss in GLI-specific binding in nuclear proteins (10 μg/lane) isolated from HeLa and SiHa cells treated with increasing concentrations of cyclopamine. NS: non-specific. Fold change was calculated following densitometric evaluation (B1: Band First, B2: Band second). **(e)** Effect of cyclopamine on viability of cervical cancer cell lines. Viability of cervical cancer cells treated with increasing concentration of cyclopamine was measured by MTT assay as described in Methods. The results are representative of three independent experiments with similar results. *p value ≤ 0.05 versus untreated control cells. ^#^*p* value ≤ 0.05 versus corresponding cyclopamine-treated HPV-negative C33a cells.

In view of active GLI signaling in cervical cancer cells, inhibition of GLI signaling was attempted by subjecting the cells to treatment of cyclopamine (10, 20, 40, 80 μM) for 24 h, a selective inhibitor that blocks GLI signaling by inhibition of Smo activity (Fig. 2a). Cervical cancer cells treated with cyclopamine (20 μM) demonstrated a decline in Smo expression but of different magnitudes in the three cell lines examined (Fig. 2b). SiHa cells were most sensitive with respect to Smo expression. A corresponding reduction in level of GLI1 transcripts was observed in cervical cancer cells (Fig. 2c). Examination of GLI DNA-binding activity in HeLa and SiHa cells treated with cyclopamine revealed inhibition of GLI DNA-binding activity in a dose-dependent manner in both the cell types tested (Fig. 2d). Loss of GLI signaling due to cyclopamine was accompanied with reduction in viability of cervical cancer cells (Fig. 2e). Interestingly, HPV-positive, HeLa and SiHa cells were found to be more sensitive to cyclopamine as compared to HPV-negative C33a cells. The 50% inhibitory concentration (IC_50_) of cyclopamine was 162.6 μM in C33a, 54.2 μM in HeLa and 41.1 μM in SiHa cells.

### Inhibition of GLI signaling in HPV-positive cervical cancer cells is accompanied with reduction in the expression level of HPV E6 oncogene

**Figure 3:**
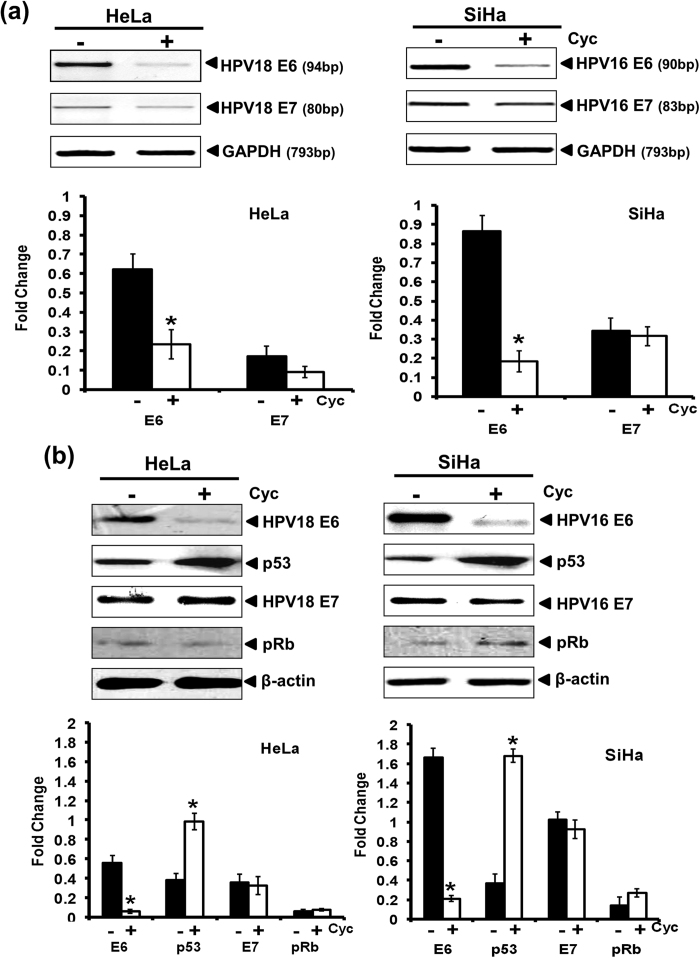
Effect of cyclopamine on the expression of HPV16 E6 and E7 oncogenes and their downstream targets. (**a**) Evaluation of E6 and E7 transcript level in cyclopamine-treated cervical cancer cells by RT-PCR. Representative photograph of EtBr-stained 2% agarose gel showing PCR amplification of E6 and E7 in cDNA prepared from cyclopamine (20 μM) treated and untreated HeLa and SiHa cells. GAPDH was used as input control for normalization. (a, lower panel) Aggregated mean (±S.D.) abundance ratios of the transcripts w.r.t. GAPDH in three independent experiments. *p value ≤ 0.05 versus untreated control cells. **(b)** Representative immunoblots showing expression of HPV16/18 E6, HPV16/18 E7 and their downstream target, p53 and pRB respectively in cellular proteins (50 μg/lane) from HeLa and SiHa cells treated with cyclopamine (20 μM) for 24 h and evaluated by SDS-PAGE followed by immunoblotting. Blots were striped and reprobed for β-actin as input control. (b, lower panel) Aggregated mean (±S.D.) abundance ratios of the band intensity of E6, E7, p53 and pRb proteins in HeLa and SiHa cells normalized to β-actin in three independent experiments. *p value ≤ 0.05 versus untreated control cells.

As the effect of inhibition of GLI signaling was prominent in HPV-positive HeLa and SiHa cells, next we examined the effect of cyclopamine on expression of HPV oncogenes E6 and E7 and their downstream targets p53 and pRb respectively. HeLa and SiHa cells treated with 20 μM of cyclopamine for 24 h revealed loss of E6 transcripts however; there was little effect on the transcript level of HPV E7 oncogene (Fig. 3a). A corresponding decline in expression of E6 oncoprotein was observed whereas the level of E7 proteins remains unaffected. Declined E6 levels were accompanied by an accumulation of p53 (Fig. 3b). On the other hand the expression of pRb remains unaltered in cyclopamine-treated cells.

### Silencing of HPV16 E6 oncogene reduced the transcript level of GLI1

**Figure 4:**
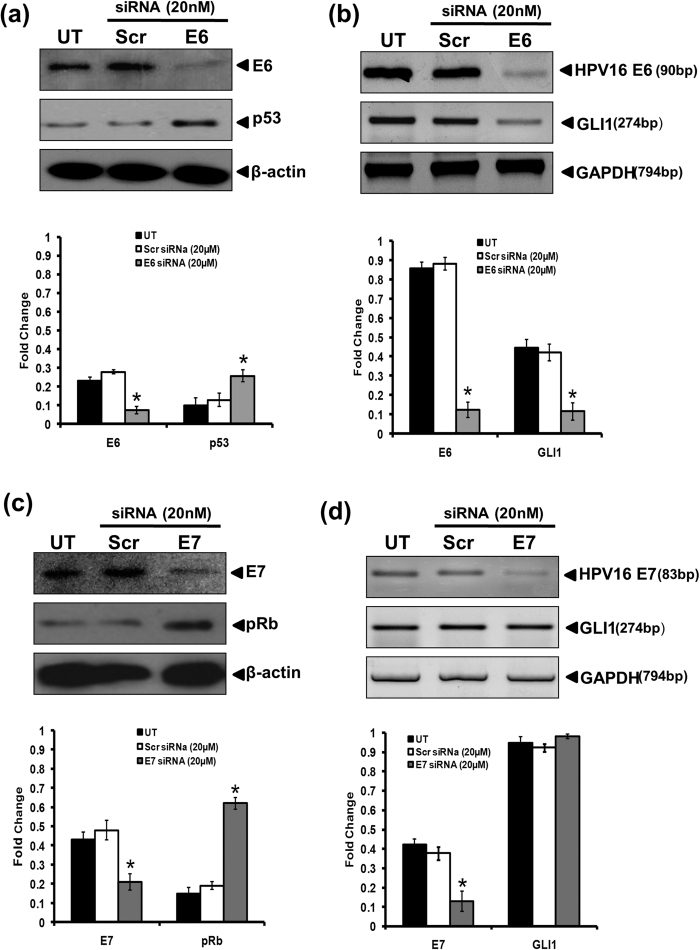
Effect of silencing HPV16 E6 or HPV16 E7 oncogene on their corresponding cellular targets and expression of GLI1. (**a**) Representative immunoblots showing expression of E6 and p53 in cellular proteins (50 μg/lane) from SiHa cells treated with HPV16 E6 siRNA (20 μM) for 48 h and evaluated by SDS-PAGE followed by immunoblotting. Blots were striped and reprobed for β-actin as input control. (a, lower panel) Aggregated mean (±S.D.) abundance ratios of the band intensity of E6 and p53 proteins in SiHa cells normalized to β-actin in three independent experiments. *p value ≤ 0.05 versus untreated control cells. **(b)** Representative photograph of EtBr-stained 2% agarose gel showing amplification of E6 and GLI1 in cDNA prepared from HPV16 E6 siRNA treated and untreated SiHa cells. GAPDH was used as input control for normalization. (b, lower panel) Aggregated mean (±S.D.) abundance ratios of the transcripts w.r.t. GAPDH in three independent experiments. *p value ≤ 0.05 versus untreated control cells. **(c)** Representative immunoblots showing expression of E7 and pRB in cellular proteins (50 μg/lane) from SiHa cells treated with specific HPV16 E7 siRNA (20 nM) for 48 h and evaluated by SDS-PAGE followed by immunoblotting. Blots were striped and reprobed for β-actin as input control. (c, lower panel) Aggregated mean (±S.D.) abundance ratios of the band intensity of E7 and pRb proteins in SiHa cells normalized to β-actin in three independent experiments. *p value ≤ 0.05 versus untreated control cells. **(d)** Representative photograph of EtBr-stained 2% agarose gel showing amplification of E7 and GLI1 in cDNA prepared from HPV16 E7 siRNA treated and untreated SiHa cells. GAPDH was used as input control for normalization. (d, lower panel) Aggregated mean (±S.D.) abundance ratios of the transcripts w.r.t. GAPDH in three independent experiments. *p value ≤ 0.05 versus untreated control cells.

To determine the functional role of HPV16 E6/E7 oncogenes in regulation of GLI signaling in cervical cancer cells, we transfected SiHa cells with HPV16 E6 or E7-specific siRNA. The efficiency of transfection as demonstrated by decline in expression level of E6 was about 83%. The inhibition of E6 oncogene was accompanied by gradual increase in the expression of p53 at protein level, demonstrating the effective silencing of E6 oncogene (Fig. 4a). Examination of the transcript level of GLI1 in HPV16 E6 siRNA-treated SiHa cells revealed a decline in the level of GLI1 transcript (Fig. 4b). On the other hand, the efficiency of E7 siRNA transfection was 78.2%. Concordant to loss of HPV16 E7, the level of pRb which is downstream target of E7 got increased in HPV16 E7-silenced SiHa cells (Fig. 4c). However, silencing of HPV16 E7 had no effect on the transcript level of GLI1 (Fig. 4d).

### Inhibition of GLI signaling results in loss of stemness in cervical cancer cells

**Figure 5:**
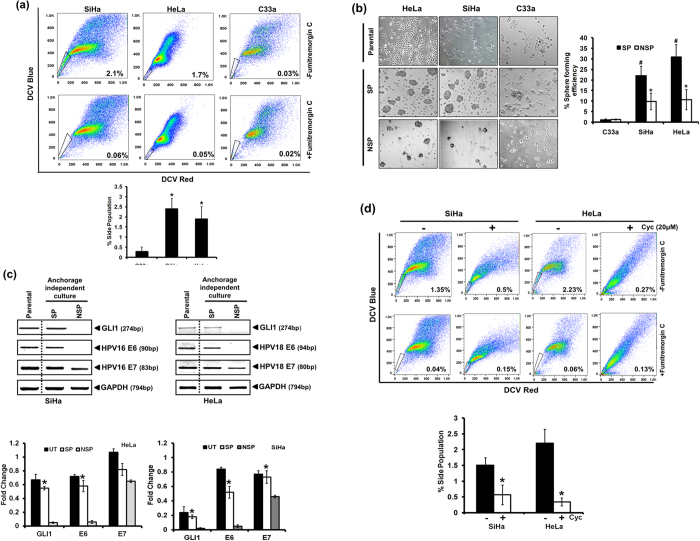
Expression of HPV E6, E7 and GLI1 proteins in cervical cancer stem cells and the effect of GLI inhibition on their proportion. (**a**) Flowcytometric analysis of DCV-stained cervical cancer cells showing the proportion of FTC-sensitive, DCV^dim^ cells having functionally active ABCG2 transporters (upper panels). SP cells were identified by back gating of FTC-sensitive DCV^dim^ population (lower panels) ran in parallel in the presence of FTC as control experiment. (a, lower panel) Cumulative data showing average number of cells in SP region in three independent experiments. Values are mean ± SD of the triplicate experiments. *p value ≤ 0.05 versus control C33a cells. (**b**) Culture of SP and NSP cells sorted from different cervical cancer cell lines and grown in anchorage-independent culture conditions. Representative picture showing the differential ability of isolated SP and NSP cells to form cervicospheres when cultured in low-adhesion substrate in keratinocyte serum free media. Each cell type was cultured as adherent control. (b, right panel) Cumulative data showing sphere-forming efficiency in three independent experiments. Values are mean ± SD of the triplicate experiments. *p value ≤ 0.05 versus control NSP cells. ^#^*p* value ≤ 0.05 versus control C33a cells. (**c**) Evaluation of GLI1, E6 and E7 transcript level in SP cells by RT-PCR. Representative photograph of EtBr-stained 2% agarose gel showing PCR amplification of GLI1, E6 and E7 in cDNA prepared from SP, NSP and parental SiHa cells. GAPDH was used as input control for normalization. (c, lower panel) Aggregated mean (±S.D.) abundance ratios of the transcripts w.r.t. GAPDH in three independent experiments. *p value ≤ 0.05 versus control NSP cells. Parental cells cultured in adherent condition were used as reference. (d) Analysis of SP cells in cervical cancer cells, SiHa and HeLa after treatment with 20 μM cyclopamine. (d, lower panel) Cumulative data showing average number of cells in SP region in three independent experiments before and after treatment with cyclopamine. Values are mean ± SD of the triplicate experiments. *p value ≤ 0.05 versus untreated control cells.

As GLI is involved in the regulation of stemness in cancer stem cells, we further examined the role of GLI signaling in cervical cancer. Side population (SP) cells were identified in cervical cancer cell lines on the basis of active ABCG2 transporters as described in Methods (Fig. 5a). The proportion of SP cells was higher in HPV-positive, SiHa and HeLa cells compared to the HPV-negative C33a cells (SP cells in SiHa −2.4 ± 0.5%, HeLa −1.9 ± 0.6% and C33a cells −0.3 ± 0.2%). The SP cells were sorted and cultured in anchorage independent conditions in Keratinocytes Serum Free Media resulted in formation of cervicospheres whereas culture with NSP cells either did not form the spheres or the size of spheres formed was small near to the cut off size (~50 cells/sphere) (Fig. 5b). The proportion of SP cells identified from HPV-negative C33a cells was small/negligible and the SP cells isolated from these cells did not form cervicospheres in anchorage independent condition. Efficient sphere formation was higher in HeLa and SiHa cells (1.2 ± 0.3% in C33a, 22 ± 4.5% in SiHa and 30 ± 5.8% in HeLa cells). The cells present in SP and NSP cultures were examined further for the transcript level of HPV E6, E7 along with the GLI1 in non-adherent culture conditions (Fig. 5c). The transcript level of GLI1 was found in an elevated state in SP cells cultured in anchorage-independent culture conditions and was comparable to their parental cells that were grown routinely in adherent state. On the other hand cells in anchorage-independent NSP cultures showed no detectable GLI transcripts. The SP cell cultures also retained their HPV E6 and E7 transcripts whereas NSP cultures of both HeLa and SiHa cells showed absence of HPV E6 and a lower level of HPV E7 transcripts. In next part, we tested the effect of inhibiting GLI signaling on proportion of the side population (Fig. 5d). HeLa and SiHa cells treated with 20 μM of cyclopamine before 24 h were subjected to SP analysis using dicycle violet (DCV)-staining followed by flowcytometric evaluation demonstrated a drastic reduction of SP cells proportion (0.56 ± 0.31% in SiHa cells treated with cyclopamine vs. 1.5 ± 0.23% in untreated SiHa cells and; 0.34 ± 0.13% in HeLa cells treated with cyclopamine vs. 2.2 ± 0.43% in untreated HeLa cells).

### Concomitant inhibition of GLI signaling along with HPV E6 oncogene effectively targeted cancer stem cells

**Figure 6:**
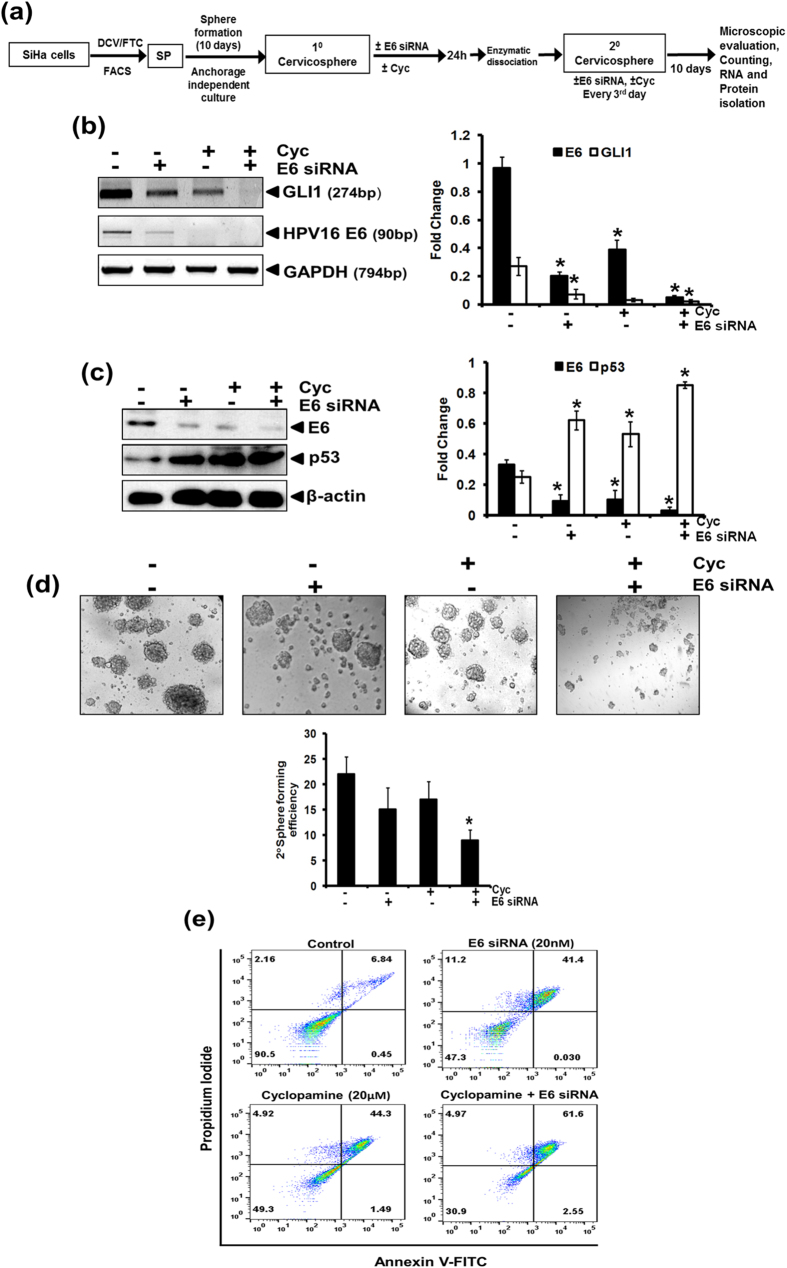
Effect of co-inhibition of E6 and GLI1 on transcript level of E6, p53, GLI and secondary cervicospheres formation ability of SP cells. (**a**) Schematic representation of the experimental design. (**b**) Evaluation of E6 and GLI1 transcript level in mRNA isolated from SiHa cells by RT-PCR. Representative photograph of EtBr-stained 2% agarose gel showing PCR amplification of E6 and GLI1 in cDNA prepared from SP cells treated with E6 siRNA (20 nM)/ cyclopamine (20 μM)/ both cyclopamine(20 μM) and E6 siRNA (20 nM)/. GAPDH was used as input control for normalization. (b, right panel) Aggregated mean (±S.D.) abundance ratios of the transcripts w.r.t. GAPDH in three independent experiments. *p value ≤ 0.05 versus control untreated SP cells. (**c**) Representative immunoblot showing expression of E6 and pRb in cellular proteins (50 μg/lane) from SP cells treated with E6 siRNA (20 nM)/ cyclopamine (20 μM)/ both cyclopamine(20 μM) and E6 siRNA (20 nM) for 48 h and evaluated by SDS-PAGE followed by immunoblotting. Blots were striped and reprobed for β-actin as input control. (c, right panel) Aggregated mean (±S.D.) abundance ratios of the proteins w.r.t. β-actin in three independent experiments. *p value ≤ 0.05 versus control untreated SP cells. (**d**) Treatment of SP cells with E6 specific siRNA/cyclopamine/or in combination. (d, lower panel) Cumulative data showing 2^0^ sphere forming efficiency in three independent experiments. Values are mean ± SD of the triplicate experiments. *p value ≤ 0.05 versus untreated control cells. (**e**) Effect of co-inhibition of E6 and GLI1 on apoptotic cell death in SiHa cells. Flowcytometric analysis of SiHa cells treated with cyclopamine, or HPV16 E6 siRNA alone or in combination for 24h. Treated cells were examined for apoptotic cells using Annexin V-FITC apoptosis detection kit.

The higher level of GLI1 and HPV E6 transcripts in stem-like cells derived from cervical cancer cell lines, prompted us to examine the sensitivity of cervicospheres formed in SP cells cultures under anchorage-independent conditions to a combined treatment of cyclopamine and E6 siRNA as per the experiment design described in Fig. 6a. The transcript level of E6 was only partly reduced in cervicospheres treated with cyclopamine or E6 siRNA alone. However, upon treatment with a combination of E6 siRNA and cyclopamine, the level of E6 transcripts was completely abolished (Fig. 6b). The loss in E6 by HPV E6 siRNA was accompanied by a strong reduction in transcript level of GLI1 and was completely absent in cells treated with cyclopamine alone or in combination with HPV16 E6 siRNA. Similar pattern of inhibition of E6 was observed in the protein level of E6 (Fig. 6c). The loss of E6 oncoproteins was associated with an increase in the level of p53 protein. Combined loss in GLI1 and E6 resulted in a severely declined sphere forming ability of cells plated in anchorage-independent conditions (Fig. 6d). Moreover, the size of spheres also reduced conspicuously to near threshold limit. In next part, we tested the effect of co-inhibition of GLI and E6 on the induction of apoptosis in these cells (Fig. 6E). The cells treated either with cyclopamine or E6 siRNA alone or in combination were stained with Annexin V- PI and subjected to flowcytometric analysis. Loss of GLI or E6 individually resulted in induction of apoptosis. However, the percentage of dead cells was higher in cells treated with the combination of cyclopamine and E6 siRNA.

### Inhibition of both GLI signaling and E6 in SiHa cells has additive effect on the cell viability

**Figure 7:**
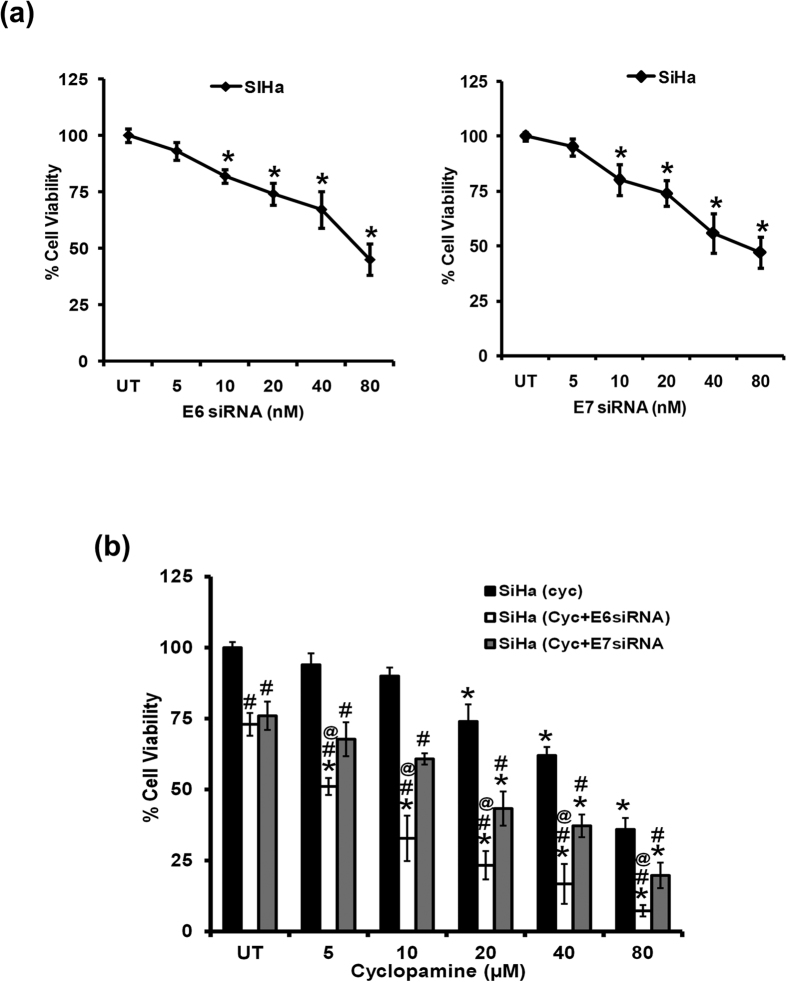
Effect of combining HPV16 E6 or E7 silencing with GLI inhibition on overall survival of cervical cancer cells. (**a**) Percent cell viability of SiHa cells treated with specific siRNA against (a, left panel) HPV16 E6 and (a, right panel) HPV16 E7 against untreated control. The results are representative of three independent experiments. *p value ≤ 0.05 versus untreated control. (**b**) Comparative analysis of percent cell viability of SiHa cells, treated with increasing concentration of cyclopamine alone in the absence or presence of siRNA against HPV16 E6 or HPV16 E7. The results are representative of three independent experiments. *p value ≤ 0.05 versus corresponding untreated cells. ^#^*p* value ≤ 0.05 versus corresponding cyclopamine-treated cells. ^@^*p* value ≤ 0.05 versus corresponding cells treated with combination of cyclopamine and E7 siRNA.

Next we examined, the effect of combined inhibition of GLI signaling along with E6 or E7 oncogenes on cell viability of adherent cell cultures of SiHa cells. SiHa cells treated with E6 or E7 specific siRNA showed decline in cell viability in a dose-dependent manner (5, 10, 20, 40 and 80 μM) as compared to untreated cells. Further, to understand the response of cyclopamine in the combination with inhibited E6 or E7 oncogene, we treated SiHa cells with 20nM concentration of specific siRNA for 24 h in the absence or presence of increasing concentration of cyclopamine. The cells co-treated with a minimum concentration of cyclopamine (5 μM) and silenced for HPV16 E6 and E7 showed a significant loss of cell viability, which increased with increasing the concentration of cyclopamine (Fig. 7). Comparable lose in cell viability was also observed in the cells treated with cyclopamine alone. However, the extent of the effect was lesser when E6 and cyclopamine were combined. Similar results were obtained in HeLa cells treated with E6 or E7 siRNA alone or in combination with cyclopamine (Supplementary Fig. 1).

## Discussion

Present study was aimed to investigate the crosstalk between HPV oncoproteins E6 and E7 with GLI signaling that regulates stemness in cancer cells. Our data demonstrate existence of an active GLI signaling operating in all the tested cervical cancer cell lines irrespective of the presence of HPV. Specific inhibition of GLI signaling by cyclopamine, or viral oncoprotein E6 by siRNA affected the expression of each other. Blocking GLI signaling along with transient silencing of E6 showed an additive effect against stemness-related functions of cancer cells under anchorage-independent conditions along with a remarkable loss of cell viability in adherent cultures.

Constitutive expression of pre-dominant GLI signaling ligands particularly Shh and Ihh in cervical cancer cells indicated existence of a potential autocrine GLI signaling loop. However, differences in the expression of downstream signaling mediators Smo, Patch, and the inhibitor Sufu was observed that correlated with variable GLI expression and activity in different cell types. HPV-positive SiHa and HeLa cells that expressed high level of Smo, Patch, GLI1 and low expression of Sufu, demonstrated a strong GLI DNA-binding activity. Though variable and detectable transcripts of all GLIs were recorded in cervical cells, our attempt to measure GLI at protein level by immunoblotting failed (data not shown). GLI proteins particularly GLI1 is a 150 kD highly unstable protein with a half-life of about 40 min^24^. However, our DNA-binding assay showed a specific binding of these proteins to their cognate consensus sequences suggesting presence of the proteins in their native form that were capable of DNA binding. Nevertheless, presence of Smo/GLI and loss of negative regulator Sufu in HPV positive cancer cells in comparison to HPV negative C33a is suggestive of an influence of HPV infection on GLI pathway. The mediators of GLI-signaling are found absent in normal tissues whereas upon carcinogenic transformation some of the key mediators are detected at abnormally high levels in cervical cancer cells^20^,^21^,^22^,^25^ and established cell lines, SiHa, Hela, Caski and C33a^23^. Not just the magnitude of the signaling molecules but also the increase in number of dysregulated pathway genes was found associated with local recurrence of late stage tumors^21^. Despite strong correlation of these markers with carcinogenic progression of cervical carcinoma, the involvement of viral oncogenes in influencing the GLI signaling remains debatable^20^,^23^,^26^ or inadequately investigated in spite of the fact that other oncogenic viruses are known to activate GLI signaling^27^. Our hypothesis further got strengthened by the results of differential response of cervical cancer cells to inhibition of GLI signaling by cyclopamine that varied with the HPV status of the cells. Cyclopamine effectively blocked Smo and reduced GLI expression and consequent DNA-binding activity in SiHa and HeLa cells and these cells had comparatively lower IC_50_ values for cyclopamine as compared to C33a. A direct binding of cyclopamine to Smo has been described^28^ however, cyclopamine has been shown to reduce Smo and GLI at protein and transcript level also^29^. The mechanism of cyclopamine-mediated transcriptional control is not known, however, loss of a positive feedback loop cannot be ruled out. Interestingly, C33a cells also responded to reduction in Smo expression but the inhibition was not translated in reduction in GLI transcripts. Apart from activation through Smo, GLI has been reported via other non-canonical pathways^19^ which might be operating in C33a cells. On the other hand, differentially higher sensitivities of HPV-positive cervical cancer cells implicate dependency of these cells on GLI signaling for their survival.

Interestingly inhibition of GLI signaling was associated with downregulation of HPV E6 oncogene and concomitant accumulation of p53. However, there was no effect on the expression of E7 or its downstream target, pRb. The specific reason underlying the selective regulation of E6 at transcript and the protein level is not understood. However, GLI has been implicated in positively influencing the AP-1 specific DNA-binding activity^30^. Worth noting, AP-1 is a key regulator of HPV oncogenic transcription that binds to early promoter and results in transcription of bicistronic E6 and E7 transcripts^31^. However, the reason why loss of GLI signaling results in a specific reduction of E6 level is not known. The bicistronic transcript undergoes differential splicing events leading to formation of different splice variants including mature transcripts of HPVE6 and E7. Loss of GLI may affect/skew these splicing events and thus prevent formation of HPVE6. However, such possibilities have not been investigated experimentally as yet. GLI belongs to zinc finger proteins that are capable of interacting with both DNA and RNA^32^ and such possibilities are not completely unexpected.

In similar set of experiments performed for specific silencing of HPV E6 or HPV E7 oncogenes in SiHa cells demonstrated a reduction in GLI1 transcripts particularly when the E6 expression was targeted using specific siRNA. A comparable siRNA-mediated loss of E7 was observed but the level of GLI1 transcripts remained unaffected. Notably, loss of E6 and E7 were accompanied by accumulation of both P53 and pRB, the corresponding targets of respective oncogenes, thus confirming the downstream physiological effects of oncogene silencing. Although, there is no report that indicates a direct control of GLI by HPV16 E6, there is a strong possibility that E6 mediates such effects indirectly via elimination of GLI1’s negative regulator p53^33^. Therefore, degradation of p53 due to HPVE6 may be a major contributor to constitutively active GLI signaling and for imparting downstream effects, leading to maintenance of stemness in the cancer cells. Characterization of cervical cancer stem-like cells by oncogene expression studies and specific silencing of E6 and E7 in the present study and earlier investigations by others^34^ demonstrated essential role of E6 but not E7 in cervical cancer stem cells that can result in a loss of cell growth and self-renewal ability, the properties which are strongly linked to GLI signaling in other epithelial malignancies^35^,^36^.

In our experimental set up, cyclopamine-mediated inhibition of GLI signaling also resulted in abrogation of stem cell properties like loss in SP cells and formation of cervicospheres in anchorage-independent conditions. Apart from HPVE6, the GLI signaling can itself override p53 by Mdm2^37^ and its inhibition by cyclopamine has been shown to restore p53 accumulation in other epithelial cancers that not related to HPV infection. Our co-inhibition studies aimed at a simultaneous targeting of both E6 and GLI in SiHa cells resulted in an additive effect on the loss of E6, GLI1 and sphere forming ability of cancer stem-like cells and also strongly affected the growth and survival of cervical cells in adherent conditions as the cells underwent apoptotic cell death. These observations, therefore, suggest a cooperative interaction between the E6 and GLI signaling.

Taken together, our data suggests a cooperative interaction between GLI signaling with viral oncoprotein E6 in HPV positive cervical cancer cells. Loss of p53 as a consequence of HPV E6 is a probable connecting link between constitutively active GLI signaling observed during persistent HR-HPV infection. On the other hand, HPVE6 via GLI signaling maintains stemness in cancer cells which could lead to progression and emergence of chemoresistance as observed in clinically-advanced cervical cancer. Therefore, targeting of both E6 and GLI can be a useful novel therapeutic strategy against cervical cancer cells that can synergistically and specifically attenuate cancer stem cells.

## Material and Methods

### Cell lines and culture conditions

The human cervical cancer cell lines, C33a (HPV-ve), HeLa (HPV18), SiHa (HPV16) and CaSki (HPV16); liver cancer cell line HepG2, breast cancer cell line (MCF-7, MDA-MB-231) and immortalized keratinocytes cell line (HaCaT) were obtained from the American Type Culture Collection (ATCC, USA). Cells (except HepG2) were maintained in Dulbecco’s Modified Eagle’s medium (DMEM), (or Minimum Essential Media (MEM) for HepG2) supplemented with 10% heat-inactivated fetal calf serum (Sigma-Aldrich Chemicals, USA), L-glutamine and 1% penicillin/streptomycin in CO_2_ incubator with a humidified atmosphere of 95% air and 5% CO_2_ at 37 °C. The authenticity of cervical cancer cell lines was periodically monitored and ascertained by HPV type-specific PCRs.

### Reagents and Antibodies

Dye Cycle Violet Stain (DCV Life Technologies, USA), Fumitremorgin C (FTC; Alexis Biochemical, Switzerland), B27, K-SFM (Invitrogen, CA) were used in the study. Specific antibodies to Shh, Ihh, Smo, Patch, Sufu, GLI1, GLI2, GLI3, HPV16/18 E6, E7, pRb, p53, β-actin (Supplementary Table 1) and specific siRNA against E6/E7 and corresponding secondary antibodies conjugated to HRP were purchased from Santa Cruz Biotechnology (USA). Smo antagonist, cyclopamine procured from Selleckchem (USA) was a gift from Prof. Ashok Kumar, University of Louisville, KY, USA. Oligonucleotides were custom-synthesized from Sigma Aldrich Chemical, U.S.A. MTT [3-(4, 5-dimethylthiazol-2-yl)-2, 5-diphenyltetrazolium bromide], penicillin-streptomycin solution, trypsin-inhibitor, TRI reagent and all other reagents were of analytical grades and were procured from Sigma-Aldrich Chemicals, (USA), unless specified.

### Immunoblotting

Immunoblotting was performed according to the protocol described earlier^38^. Total cellular proteins (50 μg/lane) were separated on 12% polyacrylamide gel and electro-transferred on PVDF membranes (Millipore Corp, USA). The membrane was blocked in PBS containing 5% non-fat skimmed milk and probed with specific antibodies against Shh, Ihh, Smo, Patch, Sufu, HPV16/18 E6, E7, p53 and pRb by incubating the membrane overnight in pre-standardized dilution of primary antibody in blocking solution at 4 °C. These blots were washed, incubated with HRP-anti-mouse IgG secondary antibodies and visualized by Luminol detection kit (Santa Cruz Biotech) by exposing the blot to KODAK X-Omat films (Kodak India, India). The Western blot membranes were stripped and reprobed for β-actin expression which was used as an internal control. The quantitative densitometric analysis of the bands was performed using my image analysis software, Thermo scientific, USA.

### Electrophoretic Mobility Shift Assay

Electrophoretic mobility shift assay (EMSA) was performed as described previously^39^. The sequence of the oligonucleotides are as follows: Forward 5′-GAT CTA AGA GCT CCC GAA GAC CAC CCA CAA TGA TGG TTG TAT GT-3′, Reverse 5′-ACA TAC AAC CAT CAT TGT GGG TGG TCT TCG GGA GCT CTT AGA TC-3^40^. Briefly, 10 μg of nuclear extract was incubated with γ-32P-radiolabeled GLI oligonucleotides for 30 min in 25 μl of reaction buffer. Protein–DNA complexes were resolved in 6% non-denaturing polyacrylamide gel (crosslinking ratio, 29:1) and exposed to phosphorimager (Bio-Rad Laboratories). To check if the observed shifted bands are specific for GLI, complexes of the GLI probe and nuclear proteins from SiHa and HeLa were competed with 100-fold molar excess of the unlabeled GLI probe prior to their loading on gel.

### Reverse Transcriptase PCR (RT-PCR)

Total cellular RNA was isolated from the cervical cancer cells, HeLa, SiHa and C33a as well as from cervical cancer stem-like cells using TRI reagent according to the manufacturer’s protocol. The quality and integrity of extracted RNA was checked spectrophotometrically and on 1.0% agarose gel. For reverse transcriptase-PCR (RT-PCR), 3 μg of total RNA was used to prepare cDNA using the Fermentas First Strand cDNA Synthesis kit (Thermo Scientific, USA) according to manufacturer’s protocol. RT-PCR was conducted in triplicate for HPV16 E6, HPV16 E7, HPV18 E6, HPV18 E7, GLI1, GLI2, GLI3 and GAPDH in a 25 μl of reaction using a set of specific primers. cDNA (1 μl) from each sample was used to perform the reaction. The PCR reaction proceeded as follows: 95 °C for 30 sec, 35 cycles including denaturation at 95 °C for 30 sec, annealing that varied in range of 56–62 °C for 30 sec, polymerization at 72 °C for 30 sec followed by final extension of 5 min at 72 °C. Primer sequence with annealing temperature is described in Supplementary Table 2. All quantifications were normalized to the level of GAPDH transcripts which was used as input control.

### SP analysis by flowcytometer using DCV labeling

Assessment of SP cells was performed in SiHa, HeLa and C33a cell lines by labeling the cells with DCV in the absence or presence of FTC, an inhibitor of ABCG2, as described previously^41^ with minor modifications. Cells (1×10^6^/ml) were suspended in pre-warmed DMEM (37 °C) containing 2% FBS and 2mM HEPES buffer (SP buffer). We ran the experiment in two parallel setup one with and other without 10 μM FTC. DCV was added to a final concentration of 10 μM. Cells were further incubated at 37 °C for 90 min., centrifuged, and re-suspended in cold SP buffer (HBSS containing 2% FBS and 2 mM HEPES buffer) and kept on ice till acquisition. To check the effect of cyclopamine on proportion of SP cells, cells were treated with cyclopamine 24 h before SP analysis. Data was collected on BD FACS ARIAIII flowcytometer using FACS Diva (BD Biosciences, USA) and analyzed using FlowJo software (TreeStar, USA). For SP and NSP gating, cells were distinguished from debris on flowcytometer based on Forward Scatter (FSC) and Side Scatter (SSC). Doublets and aggregates were gated out based on SSC area (SSC-A) versus height (SSC-H) to ensure that a detected signal arises from single cells. The DCV fluorescence was excited with violet laser at 407 nm and was measured with 450/40BP (DCV-Blue) and 565LP (DCV-Red) filters and was displayed as dual-fluorescence dot plot on a linear scale in presence or absence of FTC. Latter, a gate drawn on the limit of DCV^dim^ staining during FTC inhibition includes fewer SPs cells recognized as a dim tail extending from main population with the characteristic low fluorescence whereas intense fluorescence signal of bulk population were defined as NSP cells (DCV^bright^). Both SP and NSP cells were sorted and cultured as indicated.

### Primary cervicospheres formation

Primary cervicospheres culture was performed as described earlier^42^. Briefly, single cell suspension of SP and NSP cells obtained after sorting were centrifuged at 1,000 rpm, washed with 1 × PBS, and resuspended in 1ml of KSFM. Viability of cells was determined by trypan blue and cells (1 × 10^4^) were seeded in 6-well ultra-low attachment plates (BD, U.S.A) in Keratinocyte Serum Free Media (KSFM) supplemented with B27 (1:50 dilution). Cells were routinely observed for sphere formation. Additional 500 μl KSFM was added every 3^rd^ day. On day 10, numbers of the spheres (size of more than 50 cells) were counted and microscopic pictures were captured at 100x magnification. Sphere forming efficiency was calculated as percentage by dividing no. of sphere formed to total no. of cells seeded

### Secondary cervicospheres formation

Primary cervicospheres were treated either with cyclopamine (20 μM) or E6 specific siRNA (20 nM) or both. After 24 h of treatment, suspension of primary cervicospheres was collected in a 1.5ml microcentrifuge tube and centrifuged at 1,000 rpm. Cervicospheres were washed with 1 × PBS and were incubated with 0.25% trypsin-EDTA solution for 5 min. Trypsin was inactivated by adding 1 ml trypsin inhibitor solution. Cells were mechanically disrupted with pipette to obtain single cells and centrifuged at 1,000 rpm for 5 minutes. Trypsin inhibitor was removed from the pellet. Single cells were resuspended in KSFM and counted by trypan blue. Cells (1 × 10^4^) were again re-plated in ultra-low attachment plates and allowed to culture for 10 days. Cyclopamine (20 μM) or E6 specific siRNA (20 nM) was added every third day of culture. On day 10, secondary cervicospheres were counted and microscopic pictures were captures at 100x magnification.

### RNA interference Assay

Inhibition of HPV16 E6/E7 oncogenes was performed by transient transfection of commercially available E6/E7 specific oligonucleotides (Santa Cruz) by a method described earlier^43^. Cells (1 × 10^5^) were seeded in each well of a 6-well plate and incubated at 37 °C in a CO_2_ incubator for 24 h until the cells were ~50–60% confluent. Cells were exposed to RNAiMax transfection reagent (Invitrogen, USA) in the presence of siRNA against HPV16 E6/E7 at 20 nM concentration according to the manufacturer’s instructions. At the same time, cells were also transfected with control scrambled siRNA (20 nM). Treated cells were incubated for another 24 h for mRNA extraction. The efficiency of siRNA silencing was determined by examination of the level of the targeted transcript and protein with respect to untransfected controls by RT-PCR and immunoblotting respectively.

### Statistical analysis

The data analyses were performed using the statistical software GraphPad Prism (GraphPad Software, Inc., California). All cell culture experiments were carried out at least in three independent experimental runs. Statistical significance of difference between the two test groups was analyzed by the Student’s *t-*test and multiple comparisons versus control group were assessed by ANOVA. *p* values < 0.05 were considered significant.

## Additional Information

**How to cite this article**: Vishnoi, K. *et al*. Cross-talk between Human Papillomavirus Oncoproteins and Hedgehog Signaling Synergistically Promotes Stemness in Cervical Cancer Cells. *Sci. Rep.* **6**, 34377; doi: 10.1038/srep34377 (2016).

## Supplementary Material

Supplementary Information
