# Supplementary material for: Cross-talk between Human Papillomavirus Oncoproteins and Hedgehog Signaling Synergistically Promotes Stemness in Cervical Cancer Cells
Source: Sci Rep. 2016 Sep 28;6:34377. doi: 10.1038/srep34377 (PMC5039669; doi:10.1038/srep34377)
Supplement: Supplementary Information [file srep34377-s1.doc]

**Cross-talk between Human Papillomavirus Oncoproteins and Hedgehog Signaling Synergistically Promotes Stemness in Cervical Cancer Cells**

**Kanchan Vishnoi, Sutapa Mahata, Abhishek Tyagi, Arvind Pandey, Gaurav Verma, Mohit Jadli, Tejveer Singh, Sukh Mahendra Singh, Alok C. Bharti**

**Supplementary Methods**

**Isolation of total, cytoplasmic and nuclear proteins**

Total cellular protein for immunoblotting was isolated from cancer the method described previously [^1^](#_ENREF_1). Cells (1 x 10^6^) were collected by scraping and washed with PBS. Total proteins from the cells were prepared by incubating the cells at 4ºC for 1h in lysis buffer [20mM Tris (pH 7.4), 250 mM NaCl, 2 mM EDTA (pH 8.0), 0.1% Triton X-100, 0.01 mg/ml aprotinin, 0.005 mg/ml leupeptin, 0.4 mM PMSF, and 4 mM Na_3_VO_4_] followed by centrifugation at 14,000 rpm at 4ºC. Alternatively, for nuclear protein, Cells (1 x 10^6^) were re-suspended in ice-cold buffer A (20mM HEPES (pH 7.6), 20% (w/v) Glycerol, 10mM NaCl, 1.5mM MgCl_2_, 0.2mM EDTA, 1mM DTT, 1mM PMSF, 2mg/ml Leupeptin and 10mg/ml Aprotinin) and incubated on ice for 10 min with frequent vortexing. Lysate was centrifuged at 4,000 rpm for 10 min at 4ºC to obtain supernatant that consisted of cytoplasmic protein. The remaining pellet containing isolated nuclei was resuspended in buffer B (20mM HEPES (pH 7.6), 25% (w/v) Glycerol, 500mM NaCl, 1.5mM MgCl_2_, 0.2mM EDTA, 1mM DTT, 1mM PMSF, 2mg/ml Leupeptin and 10mg/ml Aprotinin] and centrifuged after incubation for 1h with repeated vortexing on ice at 14,000rpm for 20 min at 4ºC to obtain supernatant containing nuclear protein. The concentration of proteins isolated was determined by spectrophotometric method using Bradford dye (BioRad, Hercules, CA, USA) according to the manufacturer instructions and were stored in aliquots at -80 ºC till further use.

**MTT assay**

Cell viability after treatment with cyclopamine or E6/E7 siRNA was checked by MTT assay as described previously [^2^](#_ENREF_2). The cells were incubated in triplicate in a 96-well plate in the presence or absence of cyclopamine/siRNA in a final volume of 0.1 ml for 24h at 37°C in a CO_2_ incubator. Thereafter, 0.025 ml of MTT solution (5 mg/ml in PBS) was added to each well. After 2h incubation at 37°C, lysis buffer (20%SDS; 50% dimethylformamide) was added, and the extract was incubated overnight at 37°C for solublization of formazan crystals. The OD at 570nm was measured using a 96-well multi-scanner auto-reader (Biotek, USA) with the lysis buffer serving as blank. The percentage of viable cell was calculated using the following formula:

Percent Cell Viability = (OD of the experiment samples/OD of the control) × 100

**Calculation of IC_50_ of the drug**

IC_50_ of the drug was calculated on the basis of a scatter graph plotted in Microsoft Excel (USA) (where X axis is concentration and Y axis is % inhibition). To get the IC_50_, slop equation derived from the plot, Y=mx+c or Y=mx-c was resolved by taking Y=50. The experiments with Pearson’s coefficient of determination (r^2^) values >0.95 were considered valid for analysis.

**Flowcytometric analysis of apoptotic cell death by Annexin V - PI staining:**

Flowcytometric analysis of apoptotic cell death was done by routine lab protocol. SiHa cells were treated with cyclopamine, or HPV16 E6 siRNA alone or in combination for 24h. The cells were harvested, washed with PBS and incubated with Fluorescein isothiocynate (FITC) conjugated Annexin V and propidium iodide (PI) according to the Annexin V-FITC apoptosis detection kit (BD Biosciences) manufacturer’s instructions. The data for 10000 events was acquired by FACS and was analysed using Flowjo software (Becton-Dickinson Biosciences, San Jose, CA).

**Supplementary Table ST1: List of antibodies used in the present study**

| **S. No.** | **Antibodies** | **Clonality** | **Dilution** | **Manufacturer/Vendor** | **Catalogue No.** |
| --- | --- | --- | --- | --- | --- |
|  | Shh (C-18) | Goat polyclonal | 1:1000 | Santa Cruz | SC-1195 |
|  | Ihh (C-15) | Goat polyclonal | 1:1000 | Santa Cruz | SC-1196 |
|  | Smo (N-19) | Goat polyclonal | 1:1000 | Santa Cruz | SC-6366 |
|  | Patched (G-19) | Goat polyclonal | 1:1000 | Santa Cruz | SC-6149 |
|  | Sufu (H-300) | Rabbit polyclonal | 1:1000 | Santa Cruz | SC-28847 |
|  | HPV16 E6/18 E6 (C1P5) | Mouse monoclonal | 3:1000 | Santa Cruz | SC-460 |
|  | HPV16 E7 (TVG710Y) | Mouse monoclonal | 3:1000 | Santa Cruz | SC-264 |
|  | HPV 18E7 (N-19) | Goat polyclonal | 3:1000 | Santa Cruz | SC-1590 |
|  | p53 (DO-1) | Mouse monoclonal | 2:1000 | Santa Cruz | SC-126 |
|  | pRb | Mouse monoclonal | 4:1000 | BD Biosciences | 554164 |
|  | β Actin | Mouse monoclonal | 1:10000 | Sigma | A1978 |

**Supplementary Table ST2: List of RT-PCR primers used in the present study**

| **Gene** | **Primers** | **Annealing temperature** | **Product Size (bp)** | **Reference** |
| --- | --- | --- | --- | --- |
| HPV16 E6 | F; 5’-GTTACTGCGACGTGAGGTATATG-3’  R; 5’-GTAAATAGTGTATGTCGTATACCTAGG-3’ | 55^o^C | 90 | [^3^](#_ENREF_3) |
| HPV16 E7 | F; 5’-CCGGACAGAGCCCATTACAAT-3’  R; 5’-TGCACACACGAAACATGCGTG-3’ | 55^o^C | 83 | [^3^](#_ENREF_3) |
| HPV18 E6 | F; 5’-CAGAGGTATTTGAATTTGCATTT-3’  R; 5’-TTAGATATGTAAATACCGTACGTC-3’ | 50^o^C | 94 | [^3^](#_ENREF_3) |
| HPV18 E7 | F; 5’-ACGTGTGTGCTTTGTACGCAC -3’  R; 5’-CTGAGTCTCCTTCTTTTGCTACTTT -3’ | 50^o^C | 80 | [^3^](#_ENREF_3) |
| GLI1 | F; 5-CTCCCGAAGGACAGGTATGTAAC-3’  R; 5-CCCTACTCTTTAGGCACTAGAGTTG-3’ | 62^o^C | 274 | [^4^](#_ENREF_4) |
| GLI2 | F; 5'-CAA GGA TTC CTG CTC ATG GG-3'  R; 5'-AGT GGC TGC CGC GTA CTT-3', | 62^o^C | 472 | [^5^](#_ENREF_5) |
| GLI3 | F; 5'-ATC CAT CTC CGA TTC CTC CAT TGC-3'  R; 5'-GTA TTC TGC TGG GCT GAC TCC TG-3 | 62^o^C | 266 | [^5^](#_ENREF_5) |
| GAPDH | F; 5’-CAAGGTCATCCATGACAACTTG-3’  R; 5’-GTCCAACCACCCTGTTGCTGTAG-3’ | 45°C | 793 | [^6^](#_ENREF_6) |

**References**

1 Bharti, A. C., Donato, N. & Aggarwal, B. B. Curcumin (diferuloylmethane) inhibits constitutive and IL-6-inducible STAT3 phosphorylation in human multiple myeloma cells. *J Immunol* **171**, 3863-3871, (2003).

2 Bharti, A. C., Donato, N., Singh, S. & Aggarwal, B. B. Curcumin (diferuloylmethane) down-regulates the constitutive activation of nuclear factor-kappa B and IkappaBalpha kinase in human multiple myeloma cells, leading to suppression of proliferation and induction of apoptosis. *Blood* **101**, 1053-1062, (2003).

3 de Boer, M. A. *et al.* High human papillomavirus oncogene mRNA expression and not viral DNA load is associated with poor prognosis in cervical cancer patients. *Clin Cancer Res* **13**, 132-138, (2007).

4 Mori, Y., Okumura, T., Tsunoda, S., Sakai, Y. & Shimada, Y. Gli-1 expression is associated with lymph node metastasis and tumor progression in esophageal squamous cell carcinoma. *Oncology* **70**, 378-389, (2006).

5 Peacock, C. D. *et al.* Hedgehog signaling maintains a tumor stem cell compartment in multiple myeloma. *Proceedings of the National Academy of Sciences of the United States of America* **104**, 4048-4053, (2007).

6 Grespi, F. & Melino, G. P73 and age-related diseases: is there any link with Parkinson Disease? *Aging (Albany NY)* **4**, 923-931, (2012).

**Supplementary Figure 1**

**
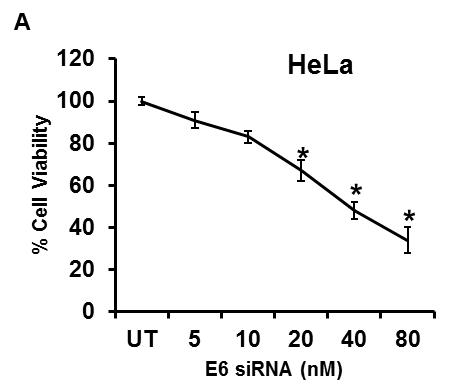

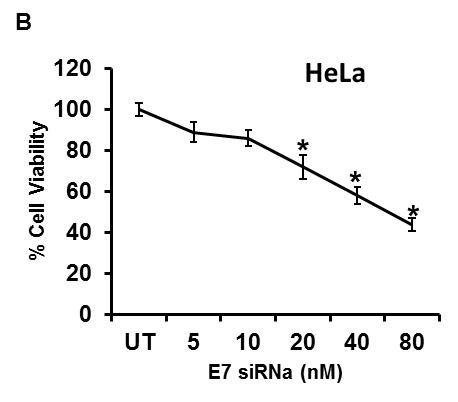
**

**
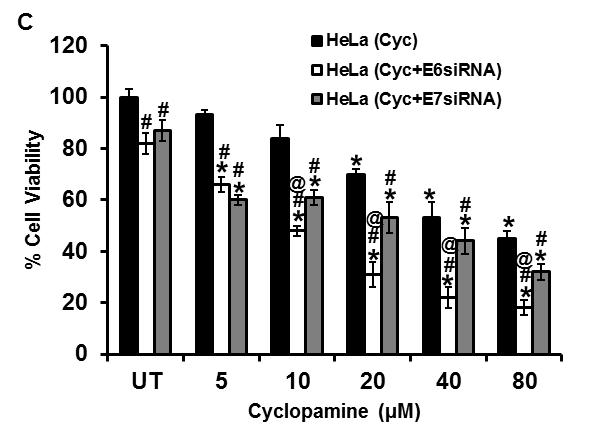
**

**SF1: Effect of combining HPV E6 or E7 silencing with GLI inhibition on overall survival of cervical cancer cells.** Percent cell viability of HeLa cells treated with specific siRNA against **A.** HPV E6 and **(ii)** HPV E7 against untreated control. The results are representative of three independent experiments. **p* value ≤ 0.05 versus untreated control. **B.** Comparative analysis of percent cell viability of HeLa cells, treated with increasing concentration of cyclopamine alone in the absence or presence of siRNA against HPV E6 or HPV E7. The results are representative of three independent experiments. **p* value ≤ 0.05 versus corresponding untreated cells. #*p* value ≤ 0.05 versus corresponding cyclopamine-treated cells. @*p* value ≤ 0.05 versus corresponding cells treated with combination of cyclopamine and E7 siRNA.
